# The Anti-Tumorigenic Mushroom *Agaricus blazei* Murill Enhances IL-1β Production and Activates the NLRP3 Inflammasome in Human Macrophages

**DOI:** 10.1371/journal.pone.0041383

**Published:** 2012-07-23

**Authors:** Tsung-Teng Huang, David M. Ojcius, John D. Young, Yi-Hui Wu, Yun-Fei Ko, Tsui-Yin Wong, Cheng-Yeu Wu, Chia-Chen Lu, Hsin-Chih Lai

**Affiliations:** 1 Center for Molecular and Clinical Immunology, Chang Gung University, Taoyuan, Taiwan, Republic of China; 2 Department of Medical Biotechnology and Laboratory Sciences, College of Medicine, Chang Gung University, Taoyuan, Taiwan, Republic of China; 3 Laboratory of Nanomaterials, Chang Gung University, Taoyuan, Taiwan, Republic of China; 4 Research Center of Bacterial Pathogenesis, Chang Gung University, Taoyuan, Taiwan, Republic of China; 5 Health Sciences Research Institute and School of Natural Sciences, University of California Merced, Merced, California, United States of America; 6 Laboratory of Cellular Physiology and Immunology, Rockefeller University, New York, New York, United States of America; 7 Biochemical Engineering Research Center, Mingchi University of Technology, Taipei, Taiwan, Republic of China; 8 Cancer Research Center, National Cheng Kung University Hospital, Tainan, Taiwan, Republic of China; 9 Department of Respiratory Therapy, Fu Jen Catholic University, Taipei, Taiwan, Republic of China; University of North Dakota, United States of America

## Abstract

*Agaricus blazei* Murill (AbM) has been reported to possess immune activity against tumors and infections through stimulation of mononuclear phagocytes. Recently, AbM extract was shown to induce the production of the pro-inflammatory cytokine, interleukin-1β (IL-1β), in human monocytes. IL-1β is a key pro-inflammatory cytokine produced by activated macrophages and monocytes and its secretion is strictly controlled by the inflammasome. The purpose of this study is to investigate the effect of AbM water extracts on the regulation of IL-1β production and activation of the NLRP3 inflammasome in human THP-1 macrophages. The NLRP3 inflammasome consists of an NLRP3 receptor, an adaptor protein called ASC, and the inflammatory protease, caspase-1. Typically, stimulation of immune cells with microbial products results in production of pro-IL-1β, but a second stress-related signal activates the inflammasome and caspase-1, leading to processing and secretion of IL-1β. Our results show that AbM enhances transcription of IL-1β and triggers NLRP3 inflammasome-mediated IL-1β secretion in human THP-1 macrophages. AbM-mediated IL-1β secretion was markedly reduced in macrophages deficient in NLRP3 and ASC, demonstrating that the NLRP3 inflammasome is essential for AbM-induced IL-1β secretion. In addition, caspase-1 was activated and involved in proteolytic cleavage and secretion of IL-1β in AbM-treated macrophages. AbM-mediated IL-1β secretion also decreased in cells treated with cathepsin B inhibitor, suggesting that AbM can induce the release of cathepsin B. Furthermore, our data show that AbM-induced inflammasome activation requires the release of ATP, binding of extracellular ATP to the purinergic receptor P2X_7_, the generation of reactive oxygen species, and efflux of potassium. Taken together, these findings reveal that AbM activates the NLRP3 inflammasome via multiple mechanisms, resulting in the secretion of IL-1β.

## Introduction

The medicinal mushroom *Agaricus blazei* Murill (AbM), a member of the *Basidiomycetes* family, is an edible mushroom that grows wildly in the coastal Piedade area of São Paulo, Brazil. It has recently received great attention in folk medicine due to its use in the prevention of a variety of diseases, including cancer, chronic hepatitis, diabetes, arteriosclerosis and hyperlipidaemia [1]. *Agaricus blazei* Murill is particularly rich in proteoglucans and different forms of β-glucans, such as β (1,3)-, β (1,4)- and β(1,6)-D-glucans [2], [3]. These β-glucans exhibit potent anti-tumor activity in mouse models and cancer cell cultures [4]–[6], and have immunomodulatory effects on monocytes, macrophages and NK cells [7]–[9]. Other reports found β-glucans from yeast and fungus also can protect host against certain types of bacterial infections in mice; these microorganisms include *Mycobacterium bovis* [10] and *Streptococcus pneumonia* [11]. An extensive study by Bernardshaw et al., (2005) showed that treatment with water-extracted AbM decreased bacteraemia and thereby increased the survival rate of mice when the mice were intraperitoneally infected with *Streptococcus pneumonia* serotype 6B [12].

Another study by Bernardshaw et al., (2005) showed that AbM induced dose-dependent production of pro-inflammatory cytokines, including IL-1β and IL-6, in human monocytes and umbilical vein endothelial cells [13]. The stimulatory effect of AbM-based extract (AndoSan™) on cytokine production (IL-1β, IL-6, IL-8, TNF-α, G-CSF and MIP-1β) in monocyte-derived dendritic cells (MDDC) was further demonstrated by Førland et al., (2010) [14]. Based on the results of gene expression microarray analysis of human monocytic THP-1 cells, Ellertsen et al., (2006) found that AbM extract strongly induced upregulation of genes for IL-1β and IL-8, but not for IL-10 and IL-12 [15]. *Agaricus brasiliensis* ( = *blazei*) extract was also found to induce mRNA expressions of TNF-α, IL-1β, and COX-2 in PMA differentiated THP-1 cells [16].

IL-1β is a key pro-inflammatory mediator crucial for local and systemic inflammation [17]. This cytokine is mainly produced by the blood monocyte, tissue macrophages and dendritic cells. B lymphocytes and NK cells can also produce IL-1β [17]. This cytokine participates in the generation of systemic and local immune responses against various strains of pathogens, and it has been implicated in the pathogenesis of inflammatory diseases, such as gout, asthma, inflammatory bowel diseases (IBDs), rheumatoid arthritis (RA), and atherosclerosis [18]–[20].

NLRP3 (also known as NALP3, cryopyrin, CIAS1, or PYPAF1), a member of the NOD-like receptor (NLR) family, was recently shown to form a cytoplasmic complex known as the NLRP3 inflammasome, which is the most fully characterized inflammasome and is responsible for activation of caspase-1 (also known as IL-1β-converting enzyme or ICE) by a vast number of pathogens and stress- or damage-related stimuli [21], [22]. Upon activation, NLRP3 recruits an adaptor, apoptosis-associated speck-like protein (ASC) containing a C-terminal caspase recruitment domain (CARD), which in turn, recruits procaspase-1 to form a multi-protein complex leading to the activation of caspase-1 via autoprocessing [23], [24]. Caspase-1 activation is required for proteolytic cleavage of pro-IL-1β, leading to the secretion of biologically active IL-1β [25]. The NLRP3 inflammasome has been also shown to be activated by endogenous or exogenous stimuli, such as ATP, monosodium urate (MSU) crystals, cholesterol crystals, UVB irradiation, pathogen-derived nucleic acids, silica, asbestos, and amyloid-β [26]–[34]. A broad array of mediators have been reported to activate the NLRP3 inflammasome. Thus, elevated reactive oxygen species (ROS) levels, depletion of intracellular potassium (K^+^), disruption of lysosomal membrane leading to the release of the cysteine protease cathepsin B, and apoptosis can modulate NLRP3 inflammasome activation [26], [30], [35], [36]. In addition, the NLRP3 inflammasome can be activated by the “danger signal”, extracellular ATP, through stimulation of the purinergic P2X_7_ receptor (P2X_7_R), which serves as an ATP-gated cation channel and rapidly causes collapse of ionic gradients by allowing K^+^ efflux [31], [37], [38].

Water extracts of AbM were previously shown to induce the production of cytokine IL-1β in human monocytic THP-1 cells; however, the molecular basis of IL-1β was not characterized. The aim of this study is therefore to investigate whether AbM stimulation of IL-1β secretion in macrophages involves caspase-1 or inflammasome activation. In the present report, we demonstrate that incubation of THP-1 macrophages with AbM extracts results in NLRP3 inflammasome-dependent activation of caspase-1 and secretion of mature IL-1β. We also investigated the molecular mechanisms involved in inflammasome and caspase-1 activation. Taken together, we found that the NLRP3 inflammasome plays an important role in regulating AbM-induced IL-1β secretion via multiple mechanisms.

## Results

### AbM extract activates pro-IL-1β expression and induces the secretion of IL-1β in human THP-1 macrophages

**Figure 1:**
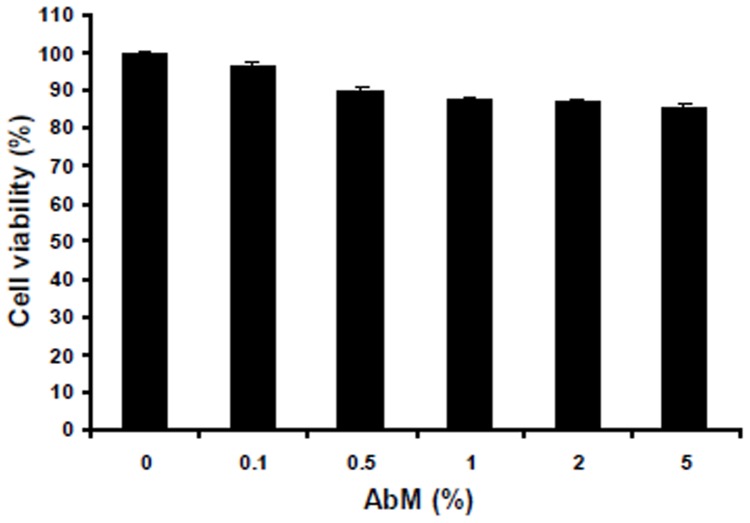
Absence of toxicity of the AbM extract on THP-1 macrophages. Cells were treated with AbM at concentrations of 0.1–5% for 24 h. Cell viability was measured by MTT assays, as described in Materials and Methods. Data are presented as means ± SE of 3 experiments preformed in duplicate.

The effects of AbM extract on the viability of human THP-1 macrophages were first studied. THP-1 macrophages were treated with AbM extract at concentrations increasing from 0.1 to 5% for 24 h at 37°C. After 24 h of incubation, the viability of THP-1 macrophages was assessed by the MTT assay. As compared to untreated control cells, the cell viability of THP-1 macrophages was slightly reduced by treatment with higher concentrations of AbM extract (Fig. 1). In addition, no striking morphological changes in the macrophages were observed during the course of this experiment.

**Figure 2:**
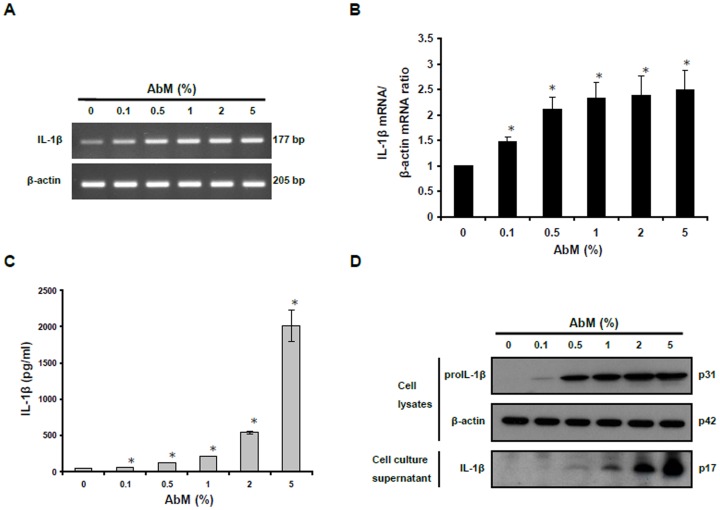
AbM extract induced secretion of IL-1β in THP-1 macrophages. (**A**) THP-1 cells were incubated with AbM at concentrations of 0.1–5% for 24 h. The mRNA expression levels of IL-1β were determined by RT-PCR analysis. (**B**) The intensity of the bands was densitometrically measured and normalized to the mRNA expression level of β-actin gene. (**C**) Cell culture supernatants were collected and assayed for IL-1β secretion by ELISA. (**D**) The presence of IL-1β in cell lysates and cell culture supernatants were analyzed by Western blot analysis. Data are presented as means ± SE of 3 experiments preformed in duplicate. *P<0.05 versus untreated cells.

AbM extract was further examined for its ability to induce the synthesis or causing the secretion of IL-1β. The macrophages were therefore treated with increasing concentrations of AbM extract for 24 h. After AbM treatment, the cells were collected and mRNA expression levels of IL-1β were analyzed by RT-PCR. The AbM extract can strongly induce expression of the IL-1β gene (Figs. 2A and 2B).

The ELISA assay was used for analyzing whether AbM extract can induce secretion of the mature form of IL-β from macrophages. Thus, the cells were treated with AbM extract (0.1–5%) for 24 h, and IL-1β was measured in the cell culture supernatants. The results show that AbM extract induced dose-dependent secretion of mature IL-1β (Fig. 2C). In order to confirm the production and processing of pro-IL-1β in the cells and also verify the secretion of mature IL-1β into the cell culture supernatants, cells treated with the AbM extract for 24 h were characterized by Western blot analysis. AbM extract enhanced proteolytic cleavage of pro-IL-1β in the cells and also induced the appearance of mature IL-1β in the cell culture supernatants (Fig. 2D). These data show that AbM extract triggers both the transcription and the secretion of IL-1β in human THP-1 macrophages.

### The secretion of IL-1β induced by AbM extract is caspase-1 dependent

**Figure 3:**
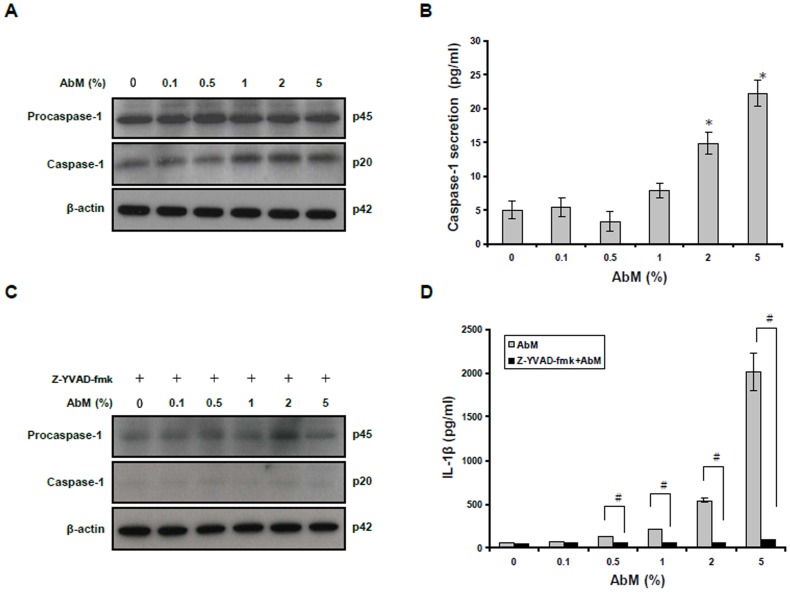
AbM extract induced caspase-1 activation in THP-1 macrophages. (**A**) and (**C**) Procaspase-1 p45 and caspase-1 subunit p20 were detected in cell lysates by Western blot analysis. (**B**) The secretion of the caspase-1 subunit p20 into the supernatants of THP-1 cells treated with AbM at concentrations of 0.1–5% for 24 h was assessed by ELISA. (**D**) THP-1 macrophages were untreated or pretreated for 30 min with the caspase-1 inhibitor, Z-YVAD-fmk (20 µM). Subsequently, cells were incubated with AbM at concentrations of 0.1–5% for 24 h, and IL-1β production was measured by ELISA. Data are presented as means ± SE of 3 experiments preformed in duplicate. *P<0.05 versus AbM-untreated control cells. ^#^ *P*<0.05 versus caspase-1 inhibitor-treated cells.

Inflammasome activation results in the recruitment and activation of caspase-1. Caspase-1 is the key enzyme involved in the processing of pro-IL-1β to form the biologically active IL-1β [39]. To characterize the activation of caspase-1, cells were treated with AbM extract for 24 h, and the presence of the inactive procaspase-1 and activated caspase-1 (p20) was measured in cell lysates by Western blot analysis. As shown in Fig. 3A, AbM extract enhanced the formation of caspase-1 p20 in human macrophages. This result was confirmed by measuring the presence of caspase-1 p20 subunits secreted into the supernatants of the AbM-treated macrophages, as detected by ELISA (Fig. 3B). The secretion of activated caspase-1 was significantly increased in the supernatants of the AbM-stimulated macrophages, indicating that AbM extract can induce the proteolytic processing of caspase-1.

In order to determine whether AbM-induced IL-1β secretion required caspase-1-mediated processing of pro-IL-1β, the macrophages were pretreated for 30 min with a specific caspase-1 inhibitor (Z-YVAD-fmk) and, subsequently, were treated with the AbM extract. The protein expression of procaspase-1 and caspase-1 in caspase-1 inhibitor-treated macrophages was determined by Western blot analysis. Caspase-1 activation was reduced dramatically in the caspase-1 inhibitor-treated cells as seen in Fig. 3C. IL-1β secretion induced by AbM extract was also significantly decreased when macrophages were pretreated with caspase-1 inhibitor (Fig. 3D). Thus, we conclude that IL-1β secretion of by AbM extract is mediated through caspase-1activation.

### AbM-induced secretion of IL-1β is dependent upon the NLRP3 inflammasome

**Figure 4:**
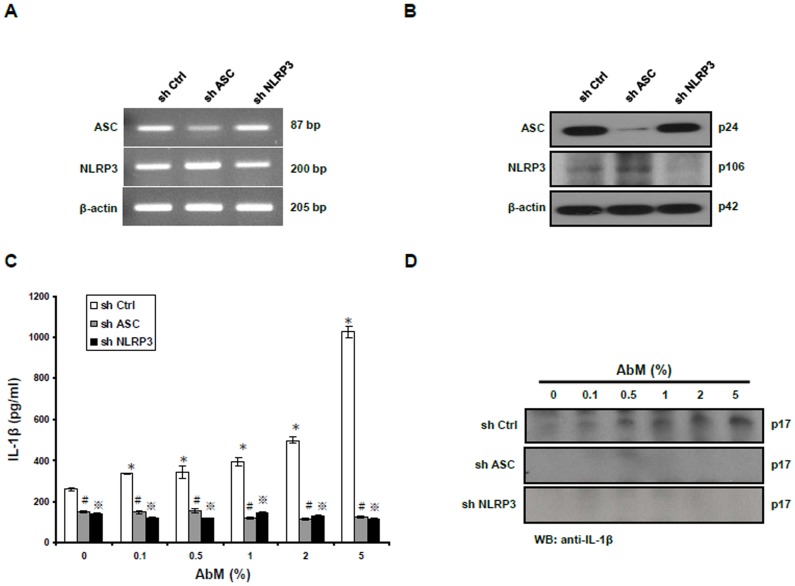
AbM-induced secretion of IL-1β depended upon the NLRP3 inflammasome. (**A**) THP-1 cells were stably transfected with shRNAs targeting ASC and NLRP3, and mRNA expression of ASC and NLRP3 was measured by RT-PCR and compared with nontarget control (shCtrl). (**B**) Knockdown cells were lysed and the protein lysates were subjected to Western blot analysis to confirm decreased protein expression of ASC and NLRP3. β-actin served as the loading control. (**C**) Knockdown THP-1 cells were treated with AbM at concentrations of 0.1–5% for 24 h, and cell culture supernatants were collected and assayed for IL-1β secretion by ELISA. (**D**) The cell culture supernatants were analyzed by Western blot analysis using anti-IL-1β antibody. Data are presented as means ± SE of 3 experiments preformed in duplicate. *shCtrl-THP-1 cells versus AbM-untreated shCtrl-THP-1 cells (*P*<0.05). ^#^shASC-THP-1 cells versus shCtrl-THP-1 cells (*P*<0.05). ^???^shNLRP3-THP-1 cells versus shCtrl-THP-1 cells (*P*<0.05).

The NLRP3 inflammasome consists of the NLRP3 receptor and an adaptor ASC, which are required for caspase-1 activation. To evaluate whether the AbM extract can activate the NLRP3 inflammasome, the role of either NLRP3 or ASC was determined by gene silencing experiments with NLRP3 or ASC shRNA in the human THP-1 macrophages. The mRNA expression level of inflammasome components was significantly reduced in comparison with nontarget shRNA, which was measured by RT-PCR (Fig. 4A). Protein depletion was also determined by Western blot analysis (Fig. 4B). The ELISA assay and Western blot analysis showed that depletion of either NLRP3 or ASC led to a significant decrease of IL-1β secretion and processing after 24 h treatment of macrophages with AbM extract (Figs. 4C and 4D). Taken together, these results demonstrate that the NLRP3 inflammasome is required for optimal AbM-induced IL-1β secretion.

### Inhibition of cathepsin B decreases AbM-induced secretion of IL-1β

**Figure 5:**
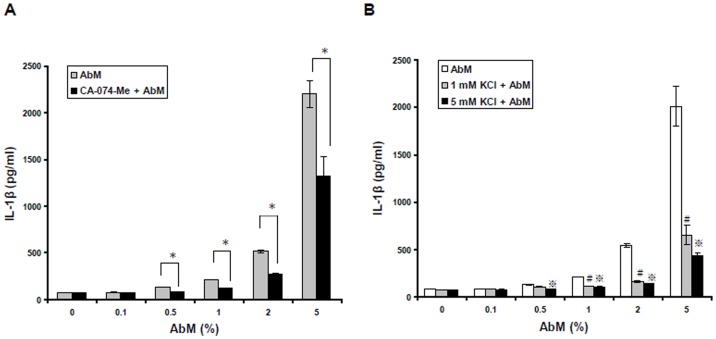
Cathepsin B and potassium efflux were required for AbM-induced inflammasome activation. Cells were untreated or pretreated for 30 min with the cathepsin B inhibitor, CA-074-Me (10 µM) (**A**), or with extracellular potassium chloride (1 mM or 5 mM) (**B**). Cells were then incubated with AbM at concentrations of 0.1–5% for 24 h. IL-1β production was measured by ELISA. Data are presented as means ± SE of 3 experiments preformed in duplicate. *P<0.05 versus cathepsin B inhibitor-treated cells. ^#^ *P*<0.05 versus 1 mM KCl-treated cells. ^???^ *P*<0.05 versus 5 mM KCl-treated cells.

Phagocytosis of MSU, silica crystals, aluminum salts, or fibrillar amyloid-β has been shown to induce lysosomal damage and the release of cathepsin B into the cytosol, leading to activation of the NLRP3 inflammasome and IL-1β secretion [30], [40]. Since Hentze et al., (2003) and Vancompernolle et al., (1998), had also shown that the lysosomal protease cathepsin B can activate caspase-1 [41], [42], we evaluated whether AbM-induced IL-1β secretion is dependent on cathepsin B. Thus, THP-1 macrophages were either untreated (control) or pretreated with cathepsin B inhibitor (10 µM CA-074-Me) for 30 min and, subsequently, treated with AbM extract at concentrations of 0.1–5% for 24 h. As seen in Fig. 5A, inhibition of cathepsin B with CA-074-Me leads to a partial but significant reduction of AbM-induced IL-1β release, implying that the effect of AbM treatment is mediated at least partially by lysosomal disruption.

### Potassium efflux contributes to AbM-induced IL-1β secretion

Lowering the intracellular concentration of potassium (K^+^) is crucial for activation of the NLRP3 inflammasome in response to a variety of stimuli [35]. In order to examine whether K^+^ efflux affects AbM-induced IL-1β secretion, THP-1 macrophages were untreated or pretreated with extracellular KCl at concentrations of either 1 mM or 5 mM for 30 min and, subsequently, treated with AbM extract at concentrations of 0.1–5% for 24 h. As seen in Fig. 5B, increasing the extracellular KCl concentration could significantly reduce AbM-induced IL-1β secretion. Our results thus suggest that K^+^ efflux is needed for AbM-mediated NLRP3 inflammasome activation in macrophages.

### Inhibition of reactive oxygen species (ROS) decreases AbM-induced secretion of IL-1β

**Figure 6:**
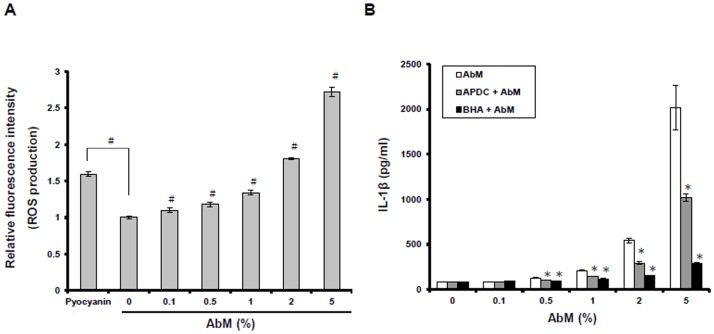
Inflammasome activation by AbM depended upon ROS production. (**A**) Cells were incubated with AbM at concentrations of 0.1–5% for 24 h, and ROS production were measured with the total ROS detection kit that finally detected by the fluorescence microplate reader. Pyocyanin (200 µM), a ROS inducer, induces the formation of ROS. (**B**) Cells were untreated or pretreated for 30 min with ROS inhibitor, APDC (50 µM) or BHA (10 µM), and then cells were incubated with concentrations of AbM at 0.1–5% for 24 h. IL-1β production was measured by ELISA. Data are presented as means ± SE of 3 experiments preformed in duplicate. ^#^ *P*<0.05 versus untreated cells. *P<0.05 versus ROS inhibitor-treated cells.

ATP and particulate activators such as asbestos and silica have been shown to trigger ROS production. The ROS production was required for downstream NLRP3 inflammasome-dependent caspase-1 activation [26], [43], [44]. Based on these previous studies, we first measured ROS production in THP-1 macrophages after exposure to AbM extract at concentrations of 0.1–5% using a commercially available ROS detection kit. A dose-dependent relationship was observed when ROS production in THP-1 macrophages was increased as increment in AbM concentration (Fig. 6A). We also analyzed whether ROS production was required for AbM-induced IL-1β secretion. THP-1 macrophages were untreated or pretreated with ROS inhibitors-APDC (50 µM) or BHA (10 µM) for 30 min and, subsequently, treated with AbM extract at concentrations of 0.1–5% for 24 h. As shown in Fig. 6B, the ROS inhibitors significantly reduced AbM-mediated IL-1β secretion in macrophages. These results suggest that AbM stimulates production of physiological levels of ROS, which result in IL-1β secretion.

### The release of ATP and binding of ATP to P2X_7_ are involved in AbM-induced IL-1β secretion

**Figure 7:**
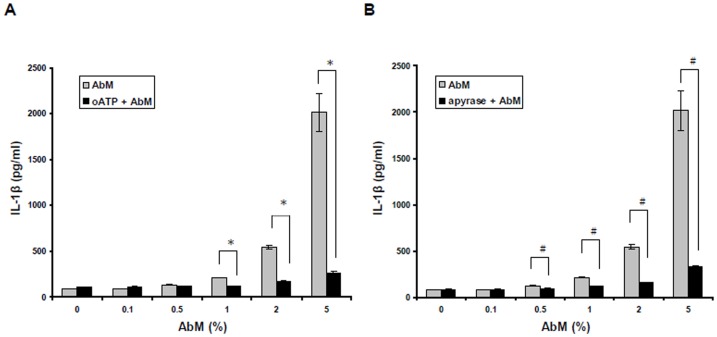
Inflammasome activation by AbM depended upon ATP release and P2X_7_R ligation. Cells were untreated or pretreated for 30 min with the P2X_7_ antagonist, oATP (250 µM) (**A**), or the extracellular ATP-hydrolyzing enzyme, apyrase (1 U/ml) (**B**); and cells were then incubated with AbM at concentrations of 0.1–5% for 24 h. IL-1β production was measured by ELISA. Data are presented as means ± SE of 3 experiments preformed in duplicate. *P<0.05 versus oATP inhibitor-treated cells. ^#^ *P*<0.05 versus apyrase-treated cells.

Ligation of the purinergic receptor P2X_7_ by extracellular ATP can stimulate NLRP3 inflammasome and caspase-1 activation the secretion of IL-1β [45], [46]. To evaluate whether the effect of AbM on IL-1β secretion is mediated through P2X_7_, we pretreated macrophages with the P2X_7_ antagonist, oxidized ATP (oATP), for 30 min, and then treated with AbM extract at concentrations of 0.1–5% for 24 h. As shown in Fig. 7A, preincubation with oATP significantly reduced the AbM-induced secretion of IL-1β, suggesting that P2X_7_R signaling is involved in AbM-induced inflammasome activation. As this result also suggested that AbM may stimulate ATP release from macrophages, we also determined the possible involvement of ATP release in AbM-induced IL-1β secretion by using apyrase, an enzyme that can rapidly hydrolyze extracellular ATP. In fact, apyrase significantly reduced AbM-induced IL-1β secretion in macrophages (Fig. 7B). Collectively, these results indicate that AbM-induced IL-1β secretion is dependent on ATP release and binding of extracellular ATP to P2X_7_.

## Discussion

AbM is thought to exert its effect mainly through modulation of the immune system, and to promote strong pro-inflammatory responses through macrophages, neutrophils and lymphocytes. In fact, modulation of the immune system has been reported to be the predominant mechanism behind the protective effect of AbM against tumor development and bacterial infection in murine models [7], [12], [47]–[49]. Previous reports have established that the expression of IL-1β mRNA was augmented by AbM extract in mouse peritoneal macrophages upon oral administration [50], and that AbM extract also upregulates the genes for IL-1β and caspase-1 in THP-1 cells [15], [16]. However, the ability of AbM to stimulate IL-1β secretion or caspase-1 activation was not investigated.

Proteolytic processing and the secretion of the mature IL-1β require the activity of caspase-1, which depends on the assembly of an inflammasome [51]. Our studies show that caspase-1 plays a major role in AbM-induced IL-1β production by macrophages. We further demonstrated that AbM-mediated caspase-1 activation and IL-1β secretion are dependent on the NLRP3 inflammasome in human macrophages.

The hallmark of NLRP3 inflammasome activation is proteolytic cleavage of cysteine protease caspase-1. Activated caspase-1 in turn cleaves pro-IL-1β into the mature form, IL-1β. Our results show that AbM stimulates cleavage of both caspase-1 and IL-1β, and that a caspase-1 inhibitor, Z-YVAD-fmk, blocks both AbM-mediated caspase-1 activation and IL-1β processing and secretion. In addition, silencing of NLRP3 and ASC decreased dramatically secretion and processing of IL-1β in response to AbM treatment.

Our results show that, besides activating the inflammasome, AbM also stimulated the transcription of IL-1β in human macrophages. These results are consistent with previous reports that AbM promotes the production of pro-inflammatory cytokines (IL-1β, IL-6, IL-8 and TNF-α), without increasing synthesis of the anti-inflammatory T-regulatory cell cytokine IL-10 or the Th1 cytokine IL-12 in human monocytes and macrophages [13], [52].

Many crystalline substances, such as silica, asbestos, and MSU crystals, have been shown to induce lysosomal destabilization and rupture, following by the release of cathepsin B into the cytosol and activation of the NLRP3 inflammasome [26], [30], [43]. A study with curdlan (fungal β-glucan) had shown that the cathepsin B inhibitor (CA-074-Me) or an inhibitor of phagocytosis (cytochalasin D) completely abrogated curdlan-induced IL-1β secretion in human macrophages [53]. Likewise, we observed that AbM-dependent NLRP3 inflammasome activation required cathepsin B activity because the cathepsin B inhibitor significantly reduced AbM-induced secretion of IL-1β in human macrophages. However, we found that cytochalasin D did not affect AbM-induced IL-1β secretion (data not shown), suggesting that AbM extracts, unlike curdlan, do not need to be internalized by the macrophages. Previous studies have demonstrated that extracellular ATP can induce cathepsin B release from lysosomal following ligation of the purinergic receptor P2X_7_ in both mouse bone marrow-derived macrophages (BMDMs) and human alveolar macrophages [54], [55]. These findings suggest that cathepsin B-dependent activation of the NLRP3 inflammasome and IL-1β secretion in AbM-treated macrophages may not be mediated by phagocytosis, but may instead be associated with the activation of P2X_7_. Moreover, cathepsin cysteine proteases are known to play an important role in the development of inflammatory diseases and cardiovascular disease [56], [57].

The NLRP3 inflammasome is activated by a wide variety of stimuli, besides cathepsin B, both K^+^ efflux and ROS production can activate the NLRP3 inflammasome [26], [35], [58]. Thus, Abdul-Sater and coworkers demonstrated that infection by an intracellular bacterial pathogen led to the efflux of intracellular K^+^, which in turn, causing ROS production and caspase-1 activation in epithelial cells [59]. Our data showed that increasing the extracellular KCl concentration or the use of ROS inhibitors significantly decreased AbM-induced secretion of IL-1β from macrophages. AbM treatment had also been previously reported to stimulate a slight but significant increase in ROS levels in granulocytes [60]. Thus, our results suggest that AbM-induced NLRP3 inflammasome activation in human macrophages is associated with K^+^ efflux and ROS production.

Finally, we demonstrated that AbM extract may induce ATP release from macrophages, since the ATP-hydrolyzing enzyme apyrase significantly reduced IL-1β secretion from AbM-treated macrophages. Pretreatment of macrophages with the P2X_7_ antagonist, oxidized ATP, also resulted in a significantly inhibition of IL-1β secretion. A previous study had shown that the extracellular release of endogenous ATP from human monocytes was an early step in the activation of the inflammasome induced by a number of pathogen- or danger-associated molecular patterns, including muramyl dipeptide (MDP) and MSU [61]. In addition, ATP treatment can induce the production of ROS, which leads to activation of caspase-1 and the secretion of IL-1β from rat alveolar macrophages [44]. In mouse BMDMs, caspase-1 activation and IL-1β secretion were induced by the addition of exogenous ATP, which activated the P2X_7_R [58]. The role of the P2X_7_R in mediating caspase-1 activation was also demonstrated by the inability of peritoneal macrophages from P2X_7_R-deficient mice to generate mature IL-1β in response to ATP [62]. These observations suggest that AbM extract treatment may stimulate the release of ATP, which activates the P2X_7_R through an autocrine loop, promoting inflammasome activation and IL-1β secretion.

**Figure 8:**
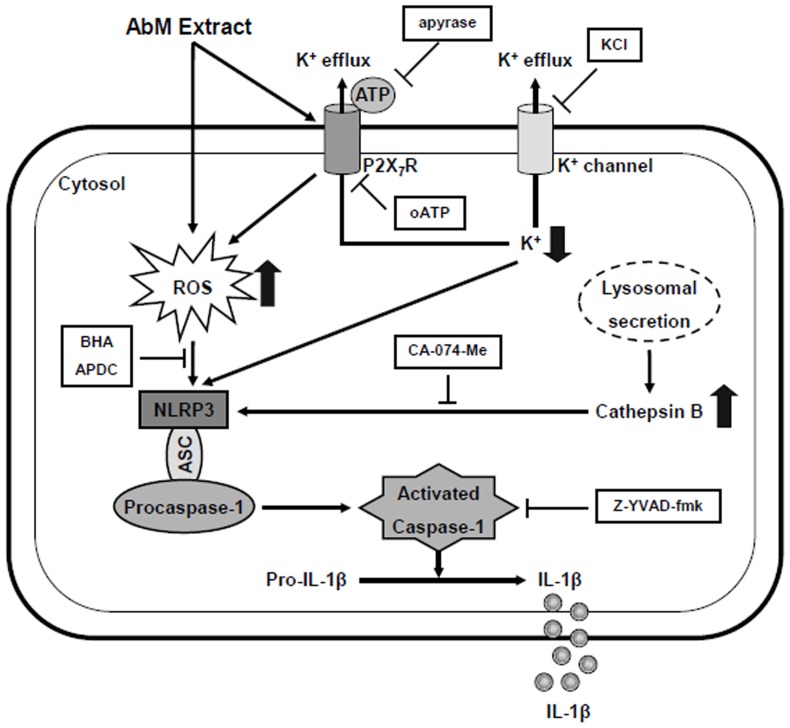
Schematic model for AbM-induced NLRP3 inflammasome activation and IL-1β secretion in human macrophages. AbM treatment resulted in ATP release and autocrine P2X_7_R activation, followed by release of cathepsin B from lysosomes, efflux of K^+^, and production of ROS. The multiple downstream pathways activated the NLRP3 inflammasome, triggering caspase-1 activation and the secretion of IL-1β.

In conclusion, our results demonstrated that AbM activated the NLRP3 inflammasome, causing caspase-1-dependent IL-1β secretion in human macrophages (Fig. 8). AbM-induced IL-1β secretion also depended upon ATP release, binding of extracellular ATP to the P2X_7_R, the release of active cathepsin B from lysosomes, K^+^ efflux, and ROS production. The production and secretion of pro-inflammatory cytokine IL-1β is crucial for stimulating innate immune responses and recruiting the phagocytic cells to defend against tumors and bacterial infection. Therefore, the findings of this study suggest that previous effects of AbM that were observed against tumors and infection may be attributed to the ability of AbM to both enhance IL-1β transcription and stimulate NLRP3 inflammasome-dependent caspase-1 activation.

## Materials and Methods

### Chemicals and reagents

Apyrase, butylated hydroxyanisole (BHA), DMSO, potassium chloride (KCl), oxidized ATP (oATP) and phorbol 12-myristate 13-acetate (PMA) were purchased from Sigma-Aldrich (St. Louis, MO). Human caspase-1 inhibitor (Z-YVAD-fmk), and (2R, 4R)-4-Aminopyrrolidine-2,4-dicarboxylic acid (APDC) were purchased from Santa Cruz Biotechnology (Santa Cruz, CA). Cathepsin B inhibitor (CA-074-Me) was purchased from Calbiochem (La Jolla, CA). Cell culture medium (RPMI 1640), FBS, penicillin and streptomycin were purchased from Gibco BRL (Grand Island, NY). For Western blot analysis, the antibodies against IL-1β was from Cell Signaling Technology (Beverly, MA); against ASC and pro-IL-1β, from Santa Cruz Biotechnology; against NLRP3, from Sigma-Aldrich; against caspase-1, from Millipore (Billerica, MA); and against β-actin, from Novus Biologicals (Littleton, CO). The secondary antibodies were horseradish peroxidase-conjugated anti-rabbit and anti-mouse IgGs (Santa Cruz Biotechnology).

### Preparation of AbM water extract

Water extracts of AbM were obtained from Chang Gung Biotechnology (Taipei, Taiwan). This mushroom extract is a commercial product and components of extract were partially released due to business confidential. The AbM mycelia powder contains per 100 g the following constituents: fat 0.48±0.12 g, sodium 66±16.5 mg, carbohydrate 49.6±12.4 g, protein 19.7±4.9 g, ash 6.48±1.62 g, sugars <16 g, ergosterol >0.039 g, β-glucan >7.62 g, water-soluble polysaccharide >11 g, and moisture <5%. Briefly, the AbM water extract was first prepared by adding 400 g of AbM mycelia powder and 10 liters of distilled water into New Brunswick Scientific BioFlo 4500 fermentor (Edison, NJ) and stirred at 121°C for 30 min with a speed of 150 rpm. The AbM mycelia extract was cooled to room temperature and subsequently, centrifuged at 4500 rpm for 30 min. The supernatants were collected and concentrated to a final volume of 2 liters by Buchi R220 vacuum concentrator (Zurich, Switzerland) at 65°C. The AbM extract was finally subjected to sterilize at 121°C for 20 min, pass by a 0.45 µm filter (Millipore), and store in dark glass bottles at −20°C until use. The amount per milliliter of the AbM extract was water-soluble polysaccharide 76 mg, adenosine 2 mg, protein 21 mg, carbohydrate 30 mg, and ash 8 mg.

### Cell culture and treatments

THP-1 (American Type Culture Collection, TIB-202), a human acute monocytic leukemia cell line, were cultured in RPMI 1640 medium supplemented with 10% (v/v) heated-inactivated FBS and 100 units/ml penicillin and 100 µg/ml streptomycin. THP-1 cells were incubated at 37°C in a 5% CO_2_ incubator with saturated humidity. For experiments, cells were plated in 6-well plates at 2×10^6^ cells per well. The cells were differentiated to adherent macrophages by overnight culture in complete medium supplemented with 500 ng/ml PMA, and then with fresh complete medium for an additional 2 days. THP-1 derived macrophages were treated with AbM at extract concentrations from 0.1 to 5% and incubated for 24 h. Cell culture supernatants were harvested at 14,000× *g*, 4°C for 5 min, and the supernatants were collected and stored at −80°C for cytokine assay. In addition, cell lysates were resuspended in the lysis buffers for RNA extraction and for Western blot analysis.

### Cell viability assay

Cell viability was determined using a commercial MTT based in vitro toxicology assay Kit (Sigma-Aldrich), which detects viable cells colorimetrically based on the purple formazan compound produced by the viable cells. THP-1 cells were initially seeded in 96-well plates (1×10^5^ cells/well) for 24 h. For macrophage differentiation, cells were treated and incubated with PMA in the same manner as described above. Cell media were replaced by complete media containing different concentrations of AbM extract in a range from 0.1 to 5% and then incubated with cells for 24 h. After incubation, 10 µl of MTT (5 mg/ml) were added to each well, and the plates were incubated at 37°C for 4 h. After incubation, each well was followed by eluting and dissolving the precipitate with 100 µl of the MTT solubilization solution. Cell viability was obtained by calculating absorption values at 570 nm using a VersaMax microplate ELISA reader (Sunyvale, CA). All treated samples and controls were tested in triplicate.

### ELISA

THP-1 macrophages (2×10^6^ cells/well) in 6-well culture plates were treated with AbM extracts in 1 ml of complete medium for 24 h. Cell culture supernatants were harvested as previously described. Levels of secreted IL-1β and activated caspase-1 in cell culture supernatants were measured using sandwich enzyme immuno assays (ELISA). Commercially available ELISA kits for human IL-1β and caspase-1 were purchased from R&D Systems (Minneapolis, MN), and were performed according to the manufacturer's instructions.

### Measurement of ROS production

Total ROS/Superoxide detection kit (Enzo Life sciences, Farmingdale, NY) was used for assessment of ROS production in THP-1 macrophages. Briefly, cells were first seeded (1×10^5^ cells/well) in 96-well culture plates for 24 h. For macrophage differentiation, cells were treated and incubated with PMA in the same manner as described above. Cell media were replaced by complete media containing different concentrations of AbM extract (0.1 to 5%) and then incubated for 24 h. In addition, cells were treated with ROS inducer pyocanin (200 µM), a positive control, at 37°C for 30 min. After treatment, cells were washed with 200 µl of 1× wash buffer and loaded with 100 µl of ROS/Superoxide detection reagents, and then incubated at 37°C for 1 h. Read the plates using a VersaMax microplate ELISA reader (Sunyvale, CA) at 520 nm after excitation at 488 nm. The increase in relative fluorescence intensity (RFI) was used to determine intracellular ROS production.

### Western blot analysis

Twenty-four hours after AbM extract treatment, cell extracts and cell culture supernatants were analyzed by Western blot analysis. The AbM-treated cells were collected and washed twice with PBS before incubation in RIPA lysis buffer (50 mM Tris-HCl (pH 7.4), 150 mM NaCl, 0.25% deoxycholic acid, 1% Nonidet P-40, 1 mM EDTA) (Millipore) and complete protease inhibitor cocktail (Roche, Mannheim, Germany) on ice for 30 min. Cell suspensions were then centrifuged at 15,000× g, 4°C for 30 min, and the supernatants of cell suspensions were collected and stored at −80°C. Total protein concentration in samples was determined using the Bio-Rad Bradford assay (Herculus, CA). Protein profiles were separated by electrophoresis in 10 to 15% SDS-polyacrylamide gels and transferred onto Millipore PVDF membranes, and specific proteins were detected by the appropriate primary and secondary antibodies before visualization using an enhanced chemiluminescence detection kit (Millipore).

### RNA isolation and RT-PCR

Total RNA was extracted from THP-1 cells using total RNA mini kit, and RNA extraction was followed by the Geneaid's instruction (Taipei, Taiwan). Two micrograms of RNA were reversely transcribed reaction volumes of 20 µl which contains an oligo (dT) primer (Invitrogen, Carlsbad, CA) and the M-MLV reverse transcriptase (Promega, Madison, WI). The cDNA for ASC, IL-1β, NLRP3 and β-actin were amplified by PCR with specific primers: ASC forward primer 5′-ATCCAGGCCCCTCCTCAGT-3′, and reverse primer 5′-GTTTGTGACCCTCCGCGATAAG-3′; IL-1β forward primer 5′-AAAAGCTTGGTGATGTCTGG-3′, and reverse primer 5′-TTTCAACACGCAGGACAGG-3′; NLRP3 forward primer 5′-CTTCTCTGATGAGGCCCAAG-3′, and reverse primer 5′-GCAGCAAACTGGAAAGGAAG-3′; and β-actin forward primer 5′-GAGACCTTCAACACCCCAGCC-3′, and reverse primer 5′-GGATCTTCATGAGGTAGTCAG-3′. The PCR products were electrophoresed in a 2% agarose gel and visualized by ethidium bromide staining on an image system.

### Generation of THP-1 cells stably expressing shRNA

THP-1 cells stably expressing short hairpin RNA (shRNA) against ASC, NLRP3, and nontarget control were obtained as previously described [63]. ASC, NLRP3, and nontarget control knockdown THP-1 cells were confirmed by RT-PCR and Western blot analysis. For experiments, cells were either untreated or treated with AbM extract in the same manner as previously described. Cell culture supernatants were harvested and stored at −80°C for cytokine assays and Western blot analysis.

### Statistical analysis

Triplicate data in each experiment were presented as mean ± SE. Mean comparisons between AbM extract-untreated control cells and treated cells were analyzed using Student's *t*-test. *P* values below 0.05 were considered statistically significant.
